# The short-term follow-up of patients with diabetes mellitus presenting with COVID-19

**DOI:** 10.25122/jml-2025-0027

**Published:** 2025-02

**Authors:** Delia-Andreea Lespezeanu, Alin Kraft, Cosmin Moldovan, Dan Ungureanu, Nicolae Bacalbasa

**Affiliations:** 1 Ion Pavel Diabetes Center, Prof. Dr. N. C. Paulescu National Institute of Diabetes, Nutrition and Metabolic Diseases, Bucharest, Romania; 2 Doctoral School, Titu Maiorescu University of Bucharest, Romania; 3 Department of General Surgery, Regina Maria Military Emergency Hospital, Brasov, Romania; 4 Department of Medical-Surgical and Prophylactic Disciplines, Faculty of Medicine, Titu Maiorescu University, Bucharest, Romania; 5 Department of General Surgery, Witting Clinical Hospital, Bucharest, Romania; 6 Department of Surgery, Carol Davila University of Medicine and Pharmacy, Bucharest, Romania; 7 Department of Visceral Surgery, Center of Excellence in Translational Medicine, Fundeni Clinical Institute, Bucharest, Romania; 8 Department of Visceral Surgery, Center of Digestive Diseases and Liver Transplantation, Fundeni Clinical Institute, Bucharest, Romania

**Keywords:** COVID-19, diabetes mellitus, outcome, inflammatory markers, Empagliflozin, interleukin-1, TNF-alpha, interleukin-6, glycemia

## Abstract

The COVID-19 pandemic has disproportionately affected individuals with diabetes mellitus (DM), significantly increasing their risk of adverse outcomes. This retrospective study aimed to explore the underlying factors contributing to the heightened vulnerability of individuals with DM to severe COVID-19. We reviewed medical records of patients diagnosed with DM from August 2020 to August 2022 and identified 60 equally divided into two groups. Group A (*n* = 30) included those diagnosed with an associated COVID-19 infection, while Group B (*n* = 30) served as the control group without a COVID-19 infection. Inflammatory biomarkers, venous blood glucose levels, and other parameters were assessed at hospital admission and again after a 14-day treatment period. Statistical analysis confirmed a strong association between diabetes and COVID-19 infection. In COVID-19 patients treated with Empagliflozin, correlations were observed between IL-1, TNF-alpha, IL-6, and blood glucose levels. Patients in Group B did not show significant improvements in inflammatory markers or blood glucose control. In contrast, in the first group, better correlations between interleukin levels and blood glucose were demonstrated, suggesting a higher success rate for that treatment. Our findings indicate that while Empagliflozin had limited efficacy in managing long-term diabetes-related complications, it might offer significant benefits in the acute phase of illness.

## INTRODUCTION

The United States has reported over 103 million confirmed COVID-19 cases and around 1,100,000 deaths. In comparison, Romania has confirmed a total of 3,346,046 COVID-19 cases, with 67,736 deaths reported [1]. Older age and underlying medical conditions, such as diabetes, hypertension, cardiovascular disease, obesity, and chronic lung disease, are commonly seen in severe COVID-19 cases [2]. Although the precise risk these conditions pose for hospitalization remains uncertain due to their prevalence in the general population [3], the pandemic disproportionately affects individuals with diabetes mellitus (DM). Over 40% of hospitalized COVID-19 patients have DM, marking it a significant risk factor for adverse outcomes [4].

DM is associated with chronic inflammation, insulin resistance, and hyperglycemia, which can trigger stronger immune responses, leading to cytokine release, hyperglycemic spikes, and further inflammation [5]. This contributes to multiorgan damage and higher mortality in COVID-19 patients [6]. Understanding the interplay between DM, inflammation, and hyperglycemia is vital for improving outcomes.

To address this issue, we conducted an observational study on individuals admitted specifically for COVID-19, with inflammatory biomarkers measured upon admission. Our study aimed to clarify why diabetes mellitus is a major risk factor for severe COVID-19. The objectives were to characterize the impact of DM on COVID-19-related outcomes in relation to inflammation, identify key risk factors among patients with DM, and explore the interactions between inflammatory biomarkers, hyperglycemia, insulin therapy, and in-hospital outcomes.

## MATERIAL AND METHODS

We conducted a retrospective study at the National Institute of Diabetes, Nutrition and Metabolic Diseases Prof. Dr. N. Paulescu, a center renowned for its expertise in nutritional and metabolic disorders.

### Patient selection

We reviewed the available retrospective institutional database from August 1, 2020, to August 1, 2022, to identify eligible patients. The inclusion criteria were as follows: patients over 18 years of age, hospitalized specifically for COVID-19, with laboratory-confirmed severe acute respiratory syndrome coronavirus 2 (SARS-CoV-2) infection determined by RT-PCR testing of nasopharyngeal or oropharyngeal specimens. Patients who tested positive for SARS-CoV-2 but were hospitalized for reasons unrelated to COVID-19 were excluded. All patients were monitored until hospital discharge or death.

This process led to the identification of 60 patients with DM, who were divided into two groups: Group A (*n* = 30) patients with DM diagnosed with COVID-19, and Group B (*n* = 30), which included patients with DM without COVID-19 (control group).

### Variables and data collection

Data extraction included demographic information, medical history, and laboratory parameters. Demographic and clinical data included patient age, sex, and the type of diabetes (either type 1 or type 2). Laboratory measurements included glycated hemoglobin (HbA1c), peripheral oxygen saturation (SpO_2_), high-density lipoprotein (HDL), low-density lipoprotein (LDL), triglycerides, uric acid, urea, creatinine, and venous blood glucose levels. Inflammatory biomarkers were also evaluated, including interleukin-1 (IL-1), interleukin-6 (IL-6), D-dimer, tumor necrosis factor-alpha (TNF-α), plasminogen activator inhibitor (PAI-1), and fibrinogen. In addition, we collected data on COVID-19 treatment administered, clinical characteristics, inpatient medical therapy, hospitalization course, and outcomes. Blood samples were collected and analyzed for inflammatory biomarkers from the day of admission through to the 14^th^ day. All analyses were performed by the central laboratory at the institution where the patients were enrolled.

### Study endpoints and baseline definitions

Diabetes mellitus was defined based on a documented diagnosis in the medical records, two fasting blood glucose levels >126 mg/dl, a two-hour blood glucose level >200 mg/dl, treatment with hypoglycemic agents, or an HbA1c level of ≥ 6.5% within one year prior to admission. The estimated glomerular filtration rate (eGFR) was calculated using the Chronic Kidney Disease Epidemiology Collaboration (CKD-EPI) equation.

The primary outcome was in-hospital death, while secondary outcomes included the need for mechanical ventilation and the requirement for renal replacement therapy.

### Statistical analysis

We present the clinical characteristics of the overall cohort, stratified by diabetes mellitus (DM) history. Categorical variables are expressed as numbers and percentages, while continuous variables are presented as mean (standard deviation) and median (interquartile range) for normally and non-normally distributed data, respectively. To compare characteristics between individuals with and without COVID-19, chi-squared tests were used for categorical variables, and unpaired *t*-tests or Mann–Whitney U tests were used for normally and non-normally distributed continuous variables, respectively. The incidences of individual outcomes (in-hospital death, need for mechanical ventilation, and requirement for renal replacement therapy) were compared between individuals with and without COVID-19 at admission using chi-squared tests.

### Autocorrelation analysis method

In this study, we utilized the autocorrelation analysis method (AAM) to evaluate the relationship between time-series datasets obtained from laboratory tests. This statistical technique quantifies the degree to which a time series correlates with itself at different time intervals, thereby revealing patterns and dependencies in the data over time. Symmetry on the time axis indicates discrete variation, highlighting the existence of autocorrelation in both current and future data, as well as in identical data series. The statistical analysis aimed to generate a three-dimensional graphic representing the distribution of the analyzed data, including medical analyses from both patient groups. This graphical representation helped to assess the distribution of data, analyze relationships between statistical data from the two groups, and determine the probability density of the results. The three-dimensional graphical representation visually illustrates the distribution of all collected analyses for both groups, offering a clear overview of the variation in the amplitude of the analyzed parameters (medical tests) at admission and after treatment. Before conducting these analyses, we confirmed that the datasets followed a Gaussian (normal) distribution, ensuring they were comparable, belonged to the same domain, and were not entirely random. With these conditions met correlation analysis was applied to identify dependencies between the medical test results and evaluate the strength of these associations. Additionally, probability density function (PDF) analysis was employed to calculate the relative probability that a random variable would fall within a specific range, helping to infer the likelihood of one sample being close to another.

## RESULTS

A total of 60 patients, aged between 30 and 80 years and with a body mass index (BMI) ranging from 25 kg/m^2^ to 37 kg/m^2^, were included in this study. Of these, 30 had a confirmed diagnosis of both diabetes mellitus (DM) and COVID-19 (Group A), while 30 had DM alone (Group B). All COVID-19 infections were confirmed via RT-PCR of nasopharyngeal or oropharyngeal samples. Insulin therapy was reviewed to assess glycemic control, particularly changes in glycated hemoglobin (HbA1c) from 7.8% to 7.1%, and the relationship of these changes to cytokine markers. Demographic and clinical data for the overall cohort are summarized in Table 1 for categorical variables and Table 2 for continuous variables. Patients received various types of antiviral treatments and were discharged home without experiencing any acute complications related to hyperglycemia.

**Table 1:** Demographic and clinical characteristics – categorical variables

Categorical variables	Total number of cases (%) (*n* = 60)	Patients with COVID-19 and DM (%) (*n* = 30)	Patients with DM (%) (*n* = 30)	*P*
Gender (male)	53%	53%	53%	<0.001
**Diabetes Type**				<0.001
Type I	6%	3%	3%	<0.001
Type II	93%	96%	90%	<0.001
**Comorbidities**				<0.001
Dyslipidemia	38%	23%	53%	<0.001
Hypertension	53%	63%	43%	<0.001
Obesity	28%	26%	30%	<0.001
Anxiety	8%	6%	10%	<0.001
Heart failure	25%	43%	6%	<0.001
Diabetic polyneuropathy	28%	20%	36%	<0.001
Diabetic retinopathy	11%	3%	20%	<0.001
Peripheral arterial disease	10%	3%	16%	<0.001
Hepatic steatosis	20%	23%	16%	<0.001
Ketoacidosis	3%	6%	0	<0.001
Stroke	3%	6%	0	<0.001
Anemia	20%	23%	16%	<0.001
Renal disease	20%	10%	30%	<0.001
**In-hospital death**	1%	3%	0	<0.001
**Empiric anti biotherapy**	98%	96%	100%	<0.001
BMI				<0.001
25-29.9	53%	53%	53%	<0.001
30-34.9	46%	46%	46%	<0.001

**Table 2:** Demographic and clinical characteristics – continuous variables

Continuous variables	Total number of cases (%) (*n* = 60)	Patients with COVID-19 and DM (%) (*n* = 30)	Patients with DM (%) (*n* = 30)	*P*
Age (yrs) (median)	55	55	55	<0.001
eRFG upon admission	0.8 mg/dl	0.95 mg/dl	1.3 mg/dl	0.0798
ml/min/1.73 m^2^	98ml/min/1.73m^2^	86ml/min/1.73m^2^	56ml/min/1.73m^2^	<0.001
Glycemia – venous blood upon admission	190 mg/dl	149 mg/dl	350 mg/dl	<0.001
Inflammatory Biomarkers (median ± SD)				
Il-1	37 (172.89)	53 (115.69)	18,5 (217.75)	0.0795
Il-6	962 (1834.28)	895 (1510.51)	1340.5 (2093.71)	0.0691
TNF-alfa	165 (146.67)	188,5 (116.46)	113.5 (172.73)	0.0782
PAI-1	387,5 (215,44)	333,5 (227,47)	409 (205,33)	<0.001
BMI (Mean)	1.2%	30.5 kg/m^2^	32.5 kg/m^2^	
Overweight patients	4.65%	27.2 kg/m^2^	27.7 kg/m^2^	
Obese patients	3.85%	33.7 kg/m^2^	37 kg/m^2^	
HbA1c	8%	7.8%	8%	0.0316
SpO_2_	83%	92%	89%	<0.001

### Autocorrelation analysis method

**Figure 1:**
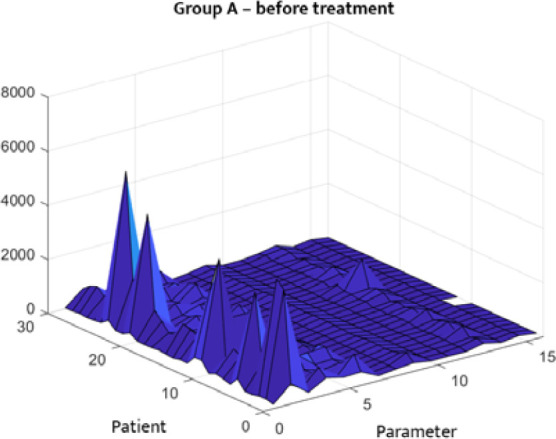
Three-dimensional correlation analysis of clinical parameters in Group A at hospital admission

**Figure 2:**
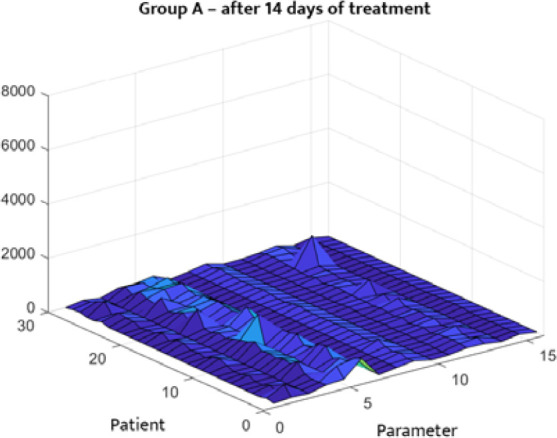
Three-dimensional correlation analysis of clinical parameters in Group A after 14 days of treatment

**Figure 3:**
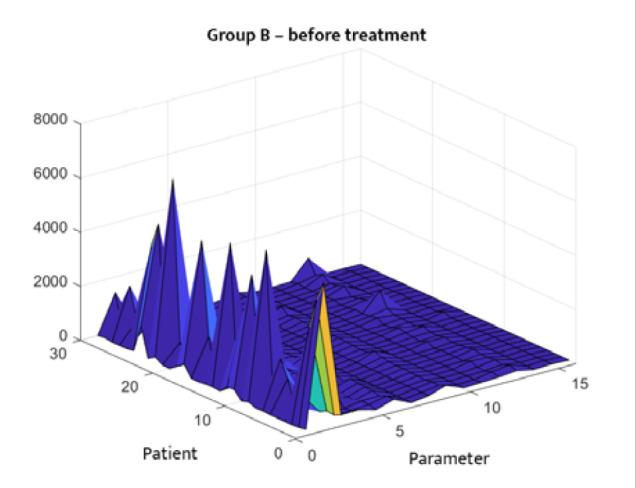
Three-dimensional correlation analysis of clinical parameters in Group B at hospital admission

**Figure 4:**
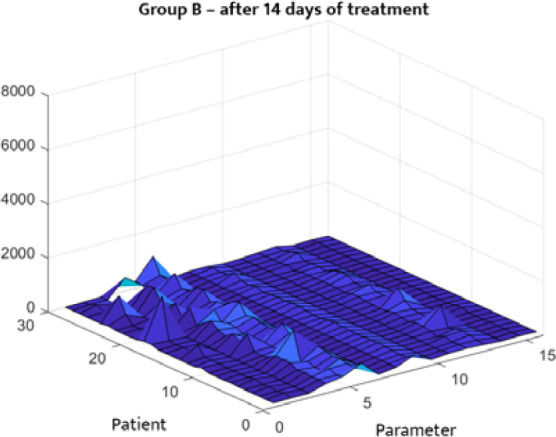
Three-dimensional correlation analysis of clinical parameters in Group B after 14 days of treatment

The statistical distribution of the analyzed clinical data is depicted in Figures 1–4. Figures 1 and 2 describe the correlations between different parameters in Group A before and after treatment. Figures 3 and 4 illustrate the parameters of Group B (patients with DM only) upon admission and on the 14^th^ day of treatment. A symmetrical pattern in these plots suggests a strong autocorrelation that does not abruptly converge toward the mean or zero.

**Figure 5:**
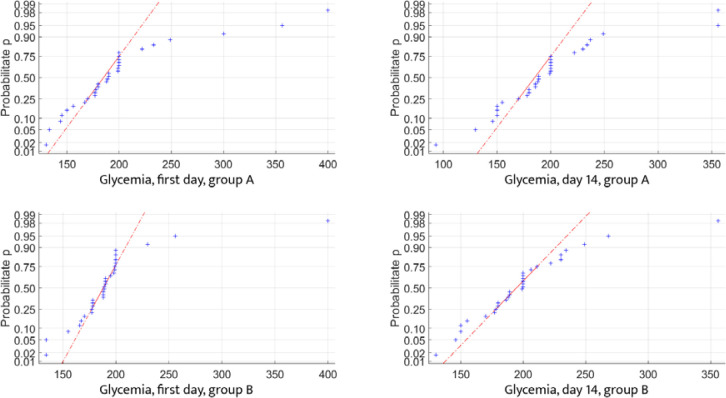
Glycemic changes in Groups A and B on Day 1 and Day 14

**Figure 6:**
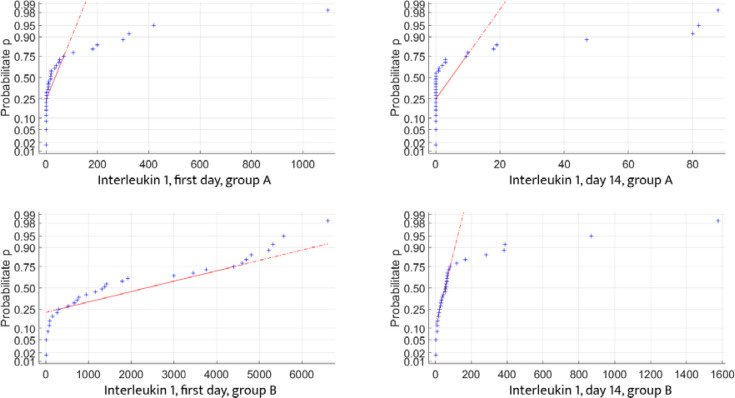
IL-1 changes in Groups A and B on Day 1 and Day 14

**Figure 7:**
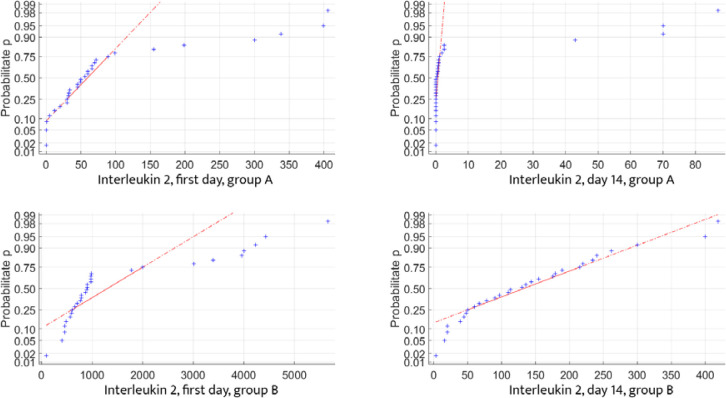
IL-2 changes in Groups A and B on Day 1 and Day 14

To ensure a high level of confidence in the autocorrelations between the analyzed data, their distribution must follow the normal distribution (Gaussian) law, as defined in mathematical statistics. We tested the distribution of inflammatory and blood glucose controls for both data sets and found that each parameter partially adhered to the normal distribution law. This partial fitting allows us to proceed with statistical calculations to determine the correlation between the data sets (Figures 5-7).

**Figure 8:**
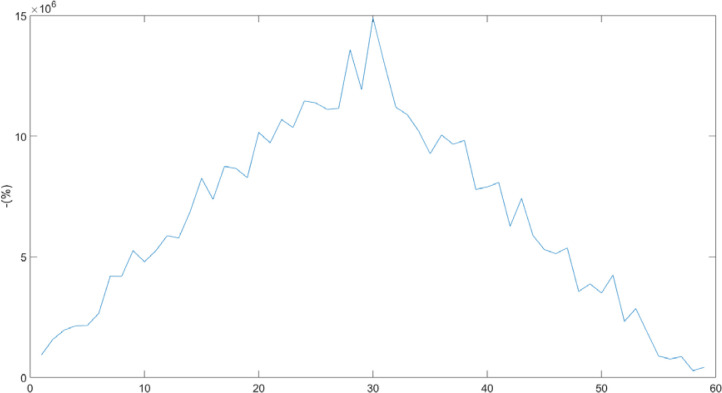
Autocorrelation between IL-6 and glycemia on Day 1

Figure 8 demonstrates a strong autocorrelation between IL-6 and blood glucose levels in Group A, as measured at the time of diagnosis. This is evidenced by the characteristic appearance, where symmetry is relative to the vertical axis, and the value does not abruptly approach zero.

**Figure 9:**
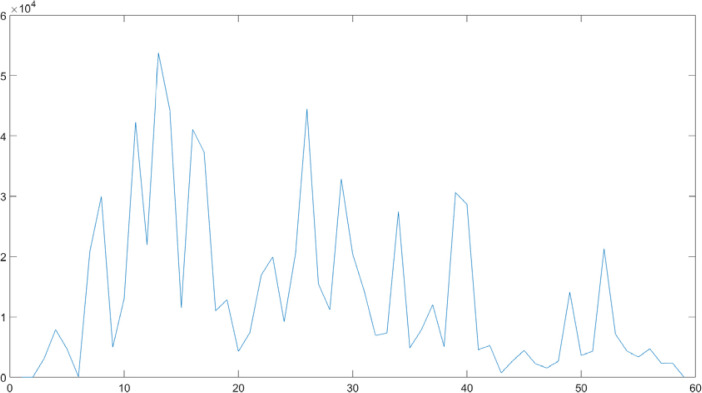
Autocorrelation of IL-1 in Group B between Day 1 and Day 14

Figure 9 illustrates the absence of autocorrelation between IL-1 levels at the time of hospitalization and on the 14^th^ day in patients from Group B. This is evident from the variation graph, which lacks an axis of symmetry.

**Figure 10:**
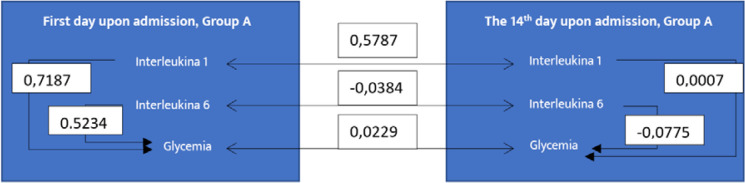
Autocorrelation coefficients for IL-1, IL-6, and glycemia in Group A

**Figure 11:**
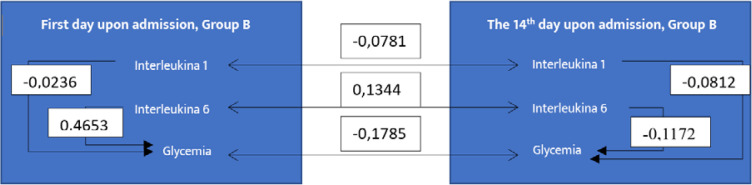
Autocorrelation coefficients for IL-1, IL-6, and glycemia in Group B

Mathematically, if the autocorrelation coefficient approaches a value of 1, it indicates a very strong correlation between the data series. Conversely, if the coefficient approaches zero, it suggests a weak autocorrelation. If the coefficient is negative, it signifies no correlation between the analyzed data [7]. We found it useful to calculate the autocorrelation coefficients between IL-1, IL-6, and glucose levels collected on Day 1 and Day 14. Figure 10 illustrates these coefficients for Group A, while Figure 11 shows them for Group B.

In Group B, we observed a very weak correlation between IL-6 levels collected on Day 1 and Day 14 (0.1344). Additionally, there was a relatively small correlation between IL-6 and blood glucose levels collected on Day 1 (0.4653), with no significant correlation between other data sets, as all values were negative. Conversely, for Group A, stronger correlations were seen between IL-1 and blood glucose (0.7187) and IL-6 and blood glucose (0.5234) on Day 1. However, by Day 14, these correlation coefficients had significantly decreased or no longer showed any correlation.

## DISCUSSION

Our results indicated that in patients with COVID-19 and DM (Group A), the correlation between IL-1 and blood glucose levels at admission was higher than at discharge, suggesting that the treatment had a positive impact on patients. In contrast, in patients with diabetes alone (Group B), no conclusions can be drawn regarding treatment success for the same parameters, as no correlation was found either at admission or discharge, with values being negative.

In Group A, the strong correlation between IL-6 and blood glucose levels at admission, which subsequently diminished during the treatment period, further supports the notion that early therapeutic interventions may effectively modulate inflammatory responses. Similar findings regarding the same parameters were observed for Group B.

The relationship between IL-1 at admission and discharge was stronger in Group A than in Group B, suggesting that the treatment administered to Group A was more effective at mitigating the inflammatory response. In Group B, most correlation coefficients were low (close to 1 or negative), except for one, suggesting that the treatment did not produce the expected results over the 14-day period, especially concerning IL-1, IL-6, and blood glucose levels.

The correlation between TNF-α and blood glucose levels also decreased significantly in Group A from admission to discharge, further supporting the success of treatment. In contrast, the correlation coefficients for the same parameters in Group B showed no change, preventing any conclusions about the treatment's effectiveness for these parameters. For treatment Group B, the TNF-α and blood glucose correlation at admission and discharge does not provide any meaningful information about the quality of treatment for these two parameters.

Both groups had an increase in the correlation between fibrinogen and blood glucose levels over the 14-day treatment period, as well as between PAI-1 and peripheral oxygen saturation (SpO_2_), suggesting that the treatments were not effective for these parameters. Statistical analysis revealed a strong association between diabetes and COVID-19 infection. Elevated levels of inflammatory cytokines and hyperglycemia were key indicators for the early detection of infectious and diabetic complications. Among patients with COVID-19 receiving Empagliflozin, a significant correlation was observed between IL-1, TNF-alpha, IL-6, and blood glucose levels. However, Empagliflozin therapy did not show significant efficacy in patients with diabetes—regardless of COVID-19 status— and this lack of effectiveness was consistent regardless of SpO2 levels at both admission and discharge. Given these findings, Empagliflozin therapy may be safely discontinued upon discharge, as its main benefits were seen in the acute phase of the illness.

The monitored parameters showed notable changes from day 1 to day 14, with particularly significant findings in patients with type 2 diabetes, pointing to the exacerbation of comorbidities. Mathematically, it was demonstrated that the correlation coefficient for certain parameter pairs (e.g., PAI-1 and SpO2) increased, indicating that the treatments had no positive effect on these inflammatory markers. In contrast, for other inflammatory parameters, such as fibrinogen and blood glucose, the correlation coefficient decreased from admission to discharge, suggesting that the therapies provided were more effective in managing these specific markers.

During the early days of hospitalization, no significant acute or chronic renal changes were observed; although serum creatinine levels were initially elevated, they tended to normalize by discharge. Despite these improvements, hyperglycemia remained challenging to manage. IL-1, fibrinogen, and venous blood glucose emerged as critical biomarkers with anti-inflammatory, antithrombotic, and immunostimulatory roles over the 14-day period, especially in patients at low risk for cytokine storms. This outcome was achieved through early, targeted therapy initiated at admission—using IL-1 to guide treatment for infectious complications and IL-6 together with TNF-α to manage inflammatory responses.

We focused on two cytokine markers, IL-1 and IL-6, which showed correlations with venous blood glucose levels. Despite ineffective treatment, there remained a risk of cytokine storms transitioning into chronic conditions. As a result, medication often continued beyond 14 days, leading to a rebound effect of untreatable hyperglycemia. COVID-19 symptoms, influenced by IL-1 and IL-6, might also occur alongside diabetes complications, although timely treatment could show elevated biomarkers in discharged patients, either without symptoms or with atypical symptoms.

In Group A, inflammatory markers decreased during treatment, but blood glucose levels remained inconsistent, which aligns with existing literature [8]. In Group B, there was a progressive decrease in initial inflammatory markers IL-1 and IL-6, as well as in venous blood glucose levels, which are correlated. IL-1 plays a significant role in the specificity of both COVID-19 infection and diabetes.

In Group A, patients with blood glucose levels exceeding 200 mg/dl had elevated levels of IL-1 and IL-6. Despite this, these patients displayed no COVID-19 symptoms after 14 days of hospitalization.

Controlling diabetes and hyperglycemia in patients with COVID-19 resulted in the normalization of IL-1 and IL-6 levels after 14 days, irrespective of glucose levels. Additionally, there was an observed improvement in inflammatory markers during the same period, even among individuals with diabetes complications. However, patients with diabetes complications showed no significant changes in cytokine levels, making it challenging to assess their response to COVID-19.

In our population, initial exposure to COVID-19 did not result in immediate lethality, complicating the prediction of relapses in complications and comorbidities. Therefore, timely and effective treatment is crucial for controlling the spread of the virus and protecting patients.

Throughout the 14-day treatment period, glucose levels were monitored alongside IL-1 and IL-6 cytokine levels. Treatment was initiated based on symptoms, with the goal of swiftly controlling them without worsening any other complications.

The analysis of inflammatory markers and hyperglycemia in COVID-19 patients revealed a correlation between IL-1 and IL-6 levels on the first day and elevated blood glucose levels. These factors play crucial roles in the immune system but present a low risk of triggering cytokine storms. However, they remain biologically significant and may increase the risk of inducing a new storm or exacerbating an existing one, influenced by factors such as sex, age, and obesity [9].

The management of hyperglycemia, with minimal risk of pulmonary complications or fatal outcomes (evidenced by low levels of fibrinogen, C-reactive protein, procalcitonin, and D-dimers), was closely associated with IL-6 levels at the time of hospitalization. This underscores the need for the prompt initiation of antiviral therapy [10].

In patients with COVID-19, particularly those with type 1 and type 2 diabetes and complications like polyneuropathy and nephropathy, the lack of antiviral treatment upon admission increases the risk of severe cytokine storms, leading to lung damage and compromised immune function [11]. Key inflammatory markers like IL-1 and IL-6 are crucial in both COVID-19 infection and the development of cytokine storms.

A correlation between IL-1, IL-6, and blood glucose exacerbates comorbidities in these patients. Interestingly, despite elevated glucose levels, there is a sudden drop in interleukin levels over time. IL-1 is pro-inflammatory, while TNF-alpha and IL-6 have anti-inflammatory roles. Inflammatory markers such as IL-1 decrease with continuous treatment, although hyperglycemia persists in some cases [12].

Diabetes patients with COVID-19 face fluctuating blood glucose levels and elevated inflammatory markers, but these often normalize by day 14. Trends in IL-1 and IL-6 can be challenging to interpret, with occasional cases of unexplained hyperglycemia without significant inflammatory response [13]. The presence of elevated inflammatory markers in these patients can pose risks, such as myocardial infarction, underscoring the need for prompt treatment in diabetic COVID-19 patients [14].

## CONCLUSION

Our study investigated the relationship between diabetes, inflammatory markers, and the outcomes of COVID-19 treatment in hospitalized patients. We compared two treatment groups, A and B, focusing on parameters such as IL-1, IL-6, TNF-alpha, fibrinogen, and blood glucose levels. Group A, treated with a specific protocol, showed strong correlations between inflammatory markers and blood glucose at admission, with improvements observed throughout treatment, indicating treatment success. In contrast, Group B exhibited weak or negative correlations, suggesting limited treatment effectiveness. We also explored the impact of Empagliflozin therapy and found it ineffective in diabetic patients with COVID-19, as it did not significantly improve inflammatory markers or glycemic control. Elevated cytokine levels and hyperglycemia were key indicators for detecting complications, but Empagliflozin provided benefits only in the acute phase of the illness. In conclusion, our findings underscore the importance of addressing both hyperglycemia and inflammation in COVID-19 treatment for diabetic patients, suggesting that Empagliflozin may not be ideal for long-term therapy. However, the main limitation of the article is related to the small number of patients included in the study; therefore, studies on larger groups are still needed to support these conclusions.

## Personal thanks

The present study is included in wide retrospective research as part of a doctoral dissertation, developed by Delia-Andreea Lespezeanu, doctoral student; it currently pends full publication and is entitled “Favorable perspectives in the anti-infective, anti-COVID-19, and anti-diabetic treatment of systemic, metabolic, and vascular complications in operable extradigestive cancers”, under the auspices of “Titu Maiorescu” Doctoral School of Medicine, Bucharest, Romania, whose doctoral supervisor is Dan Ungureanu.
